# Tuning domain wall dynamics by shaping nanowires cross-sections

**DOI:** 10.1038/s41598-020-78761-w

**Published:** 2020-12-14

**Authors:** Dora Altbir, Jakson M. Fonseca, Oksana Chubykalo-Fesenko, Rosa M. Corona, Roberto Moreno, Vagson L. Carvalho-Santos, Yurii P. Ivanov

**Affiliations:** 1 Departamento de Física, CEDENNA, Universidad de Santiago de Chile, Avda. Ecuador, 3493 Santiago, Chile; 2 Departamento de Física, Universidade Federal de Viçosa, Avda. Peter Henry Rolfs s/n, Viçosa, MG 36570-000 Brazil; 3 Instituto de Ciencia de Materiales de Madrid, CSIC, Cantoblanco, 28049 Madrid, Spain; 4 Earth and Planetary Science, School of Geosciences, University of Edinburgh, Edinburgh, EH9 3FE UK; 5 Department of Materials Science and Metallurgy, University of Cambridge, Cambridge, UK; 6 School of Natural Sciences, Far Eastern Federal University, 690950 Vladivostok, Russia

**Keywords:** Nanoscience and technology, Nanoscale devices, Magnetic devices

## Abstract

The understanding of the domain wall (DW) dynamics along magnetic nanowires is crucial for spintronic applications. In this work, we perform a detailed analysis of the transverse DW motion along nanowires with polygonal cross-sections. If the DW displaces under a magnetic field above the Walker limit, the oscillatory motion of the DW is observed. The amplitude, the frequency of oscillations, and the DW velocity depend on the number of sides of the nanowire cross-section, being the DW velocity in a wire with a triangular cross-section one order of magnitude larger than that in a circular nanowire. The decrease in the nanowire cross-section area yields a DW behavior similar to the one presented in a cylindrical nanowire, which is explained using an analytical model based on the general kinetic momentum theorem. Micromagnetic simulations reveal that the oscillatory behavior of the DW comes from energy changes due to deformations of the DW shape during the rotation around the nanowire.

## Introduction

Progress in nanofabrication enables the development of nanomagnets with different and well-controlled shapes and sizes^1–5^. Some examples are self-organized nanowires with a triangular cross-section grown on uniaxially grooved W(110) surfaces by Borca et al.^6,7^. Also FeCo nanowires with a three-dimensional structure customizable by design were grown along arbitrary crystal directions by Tao et al.^8^. In addition, 3D focused electron beam induced deposition^9–12^ has opened new perspectives in science and technology, allowing the fabrication in one single direct-write step of nanostructures with small feature sizes, complex and diverse architectures and materials. These studies confirm the possibility of manufacturing nanowires with different cross sections, and make it necessary to analyze theoretically geometry-induced effects in the magnetization properties of nanomagnets^9,13,14^. Due to an increase in the surface/volume ratio, the shape of an element at the nanoscale is a crucial factor to determine its magnetization dynamic and static properties. Therefore, much effort has been done to understand how the shape of a nanoparticle changes its magnetic properties^15–26^.

Among several issues regarding magnetism at the nanoscale, the propagation of domain walls (DW) is at the heart of technological proposals that promote the use of their dynamical properties in devices such as “race-track” memory^27^, random access memory^28^, nano-oscillators^29,30^, interconnections between spin logic gates^31^, logic devices^32,33^, and magnetic field generation^34^. From the theoretical point of view, it was shown that the geometry of the nanowire cross-section is crucial for describing the DW dynamics. Rectangular nanowires exhibit the so-called Walker limit^35,36^, which is the loss of the linearity of the DW velocity as a funciton of magnetic field when the latter exceeds a critical value, known as Walker field ($H_W$). At this threshold field, there is the transition from a DW rigid-body purely translational motion to an oscillatory translational motion with a DW rotation. Indeed, Porter and Donahue^37^ showed that when a DW is propagating along a rectangular nanowire under the Walker regime, two movements are superimposed. The first one consists of an oscillation along the wire axis, while the second is a DW rotation around the nanostripe. When the DW rotates in 180${}^{\circ }$, it exhibits a complete oscillation of the DW position along the stripe. Therefore, the rotation frequency is twice the oscillation one. The theoretical model derived by Mougin et al.^36^ also predicts that the $H_W=0$ when the DW is displacing along nanostripes with a square cross-section. Nanowires with a circular cross-section also present $H_W=0$, and the velocity of a transverse DW varies linearly with the magnetic field^38^. Nevertheless, the analysis of the DW displacement along bent nanowires with cylindrical cross-section showed that the $H_W\propto \kappa$^39–41^, where $\kappa$ is the curvature of the nanowire. In this case, the oscillating and rotational frequencies are the same. We can then conjecture that the difference between the DW dynamics along nanostructures with rectangular and circular cross-sections can be explained because of the existence of two equivalent preferred directions (DW phase) for the DW center to point while displacing along a rectangular wire. At the same time, there exists just one preferred direction in a circular bent wire^42^, and no preferential direction in a cylindrical straight wire.

Planar magnetic nanowires are highly attractive for spintronic and magnetic-based applications. However, and in addition to race-track memories^27^, it is possible to use magnetic nanowires as different components of nanoscale devices. Such applications demand a full control of the domain wall velocity under different external stimuli and magnetic parameters. Based on the above, we investigate intrinsically 3D nanostructures where DWs may oscillate around and along the nanowire. This double oscillation allows other applications of the DW dynamics, as sources of radio-frequency electromagnetic emission^39,41,43^ or nano-oscillators^29,30,44^. Therefore, because the number of preferential directions of the DW phase plays a role in the oscillation frequency, the engineering of future 3D applications demands to understand how the shape of the nanowire cross-section may influence this regime as well as the transition between it and the regime of the linear with field velocity. In this line, aiming to get a better understanding of the oscillatory phenomenon of a DW as a function of the energy minima, we explore the dynamics of a transverse DW propagating along nanowires with different polygonal cross-sections, driven by a constant external magnetic field. The performed micromagnetic simulations reveal that depending on the wire geometry, two regimes of the DW dynamics are observed: (i) a regime in which the DW displaces linearly as a function of the external magnetic field; and (ii) a regime in which the DW presents the oscillatory behavior characterized by the Walker breakdown. In this last case, the DW oscillation frequency is strongly dependent on the shape of the 3D nanowire cross-section. We have explained the first regime with a theoretical model considering the propagation of a rigid DW. The second regime is explained by considering structural changes in the DW when it is rotating around the nanowire. Therefore, our work shows clearly that the oscillatory dynamics of a DW displacing in a straight nanowire with a polygonal cross-section depends on the number of times that the DW changes its shape during one rotation around the nanowire. That is, the frequency of the backward motion of the DW is related with the number of sides of the polygon describing the nanowire cross-section. This fact implies that the assumption of a rigid DW is valid just for nanowires with cross-sections with small area and its validity eliminates the DW oscillatory motion characterizing the Walker regime in the analyzed structures. Thus, our work provides a deeper understanding of the Walker breakdown phenomena. Here we investigated the transition between DW dynamics in triangular to circular nanowires cross-sections, showing that the Walker breakdown is specifically related to the change of the domain wall shape during the DW rotation.

## Model and results

**Figure 1:**
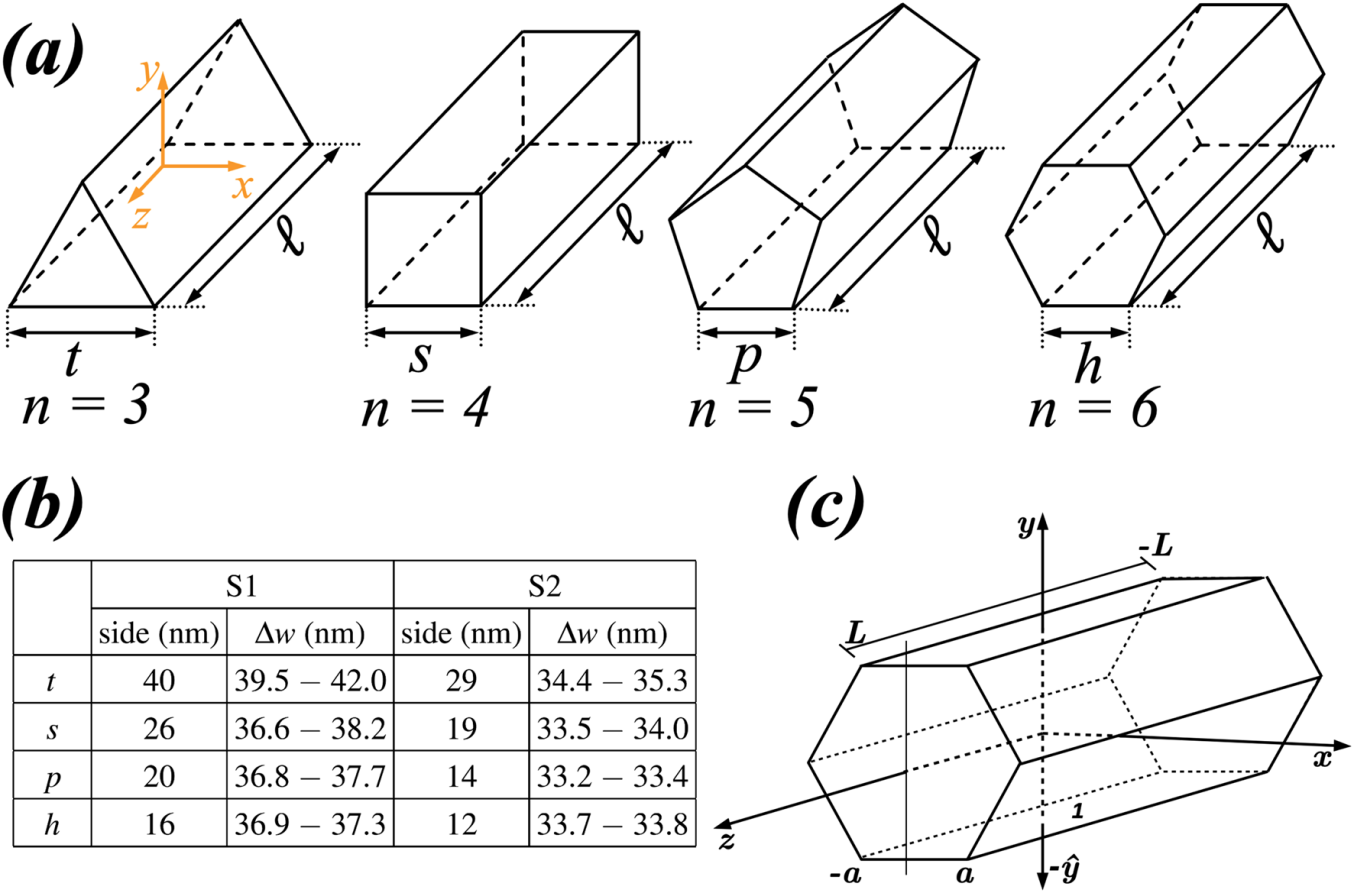
(**a**) Coordinate system and geometries of the simulated nanowires. *n* represents the number of sides of the wire cross-section. (**b**) Represent the nanowire dimensions of S1 and S2, respectively, as explained in the main text and (c) adopted coordinate of the theoretical model represented for a HNW. The number 1 indicates the polygon side representing the surface S1.

In this work, we have calculated the domain wall dynamics in nanowires with different regular cross-sections by solving the Landau–Lifshitz–Gilbert equation^45,46^ using the public micromagnetic code NMAG^47^. This open software package is based on a finite element discretization method, which results in general more suitable to describe the different shapes of cross-sections of nanostructures considered in this work. The demagnetizing field is calculated by using the hybrid finite element method/boundary element method, allowing to discretize the space only in the regions occupied by the magnetic material. The systems we study consist of nanowires of length $\ell = 1000$ nm, with regular cross-sections such as triangle (TNW), square (SNW), pentagon (PNW), and hexagon (HNW). We start considering a set (S1) of dimensions such that all nanowires have a similar cross-section areas of 700 nm$^2$ (see Fig 1b). *** The magnetic parametrization corresponds to a soft material such as permalloy to avoid screening of the effect of shaping different cross-sections into the DW dynamics by the magnetocrystalline anisotropy. Indeed, currently, the growth conditions or chemical synthesis of magnetic nanowires produce mostly polycrystalline materials^48,49^. In these circumstances it is natural first to center our study on the case with no crystalline anisotropy. We use a mesh size of 2 nm, $\mu _0 M_s=1$ T, where $M_s$ is the saturation magnetization, the exchange stiffness $A =1.3\times 10^{-11}$ J m$^{-1}$, the damping and gyromagnetic factor are $\alpha =0.01$ and $\gamma = 1.76 \times 10^{11}$ rad T$^{-1}$ s$^{-1}$ respectively, and no magnetocrystalline anisotropy is taken into account. For all the geometries, except for the square, a tail to tail transverse DW of 30 nm width is set up in the system, pointing along the positive *y*-direction (with the coordinate system as shown in Fig. 1a).*** In the square, the initial DW points along one of the lateral faces of the nanowire (in particular, we choose the positive *xy*-plane direction, as shown in the coordinate system of Fig. 1a).*** After relaxing these initial configurations for a few nanoseconds, DWs remain traverse, both placed and pointing in the same direction as before, but with different widths, corresponding to each equilibrium state.

**Figure 2:**
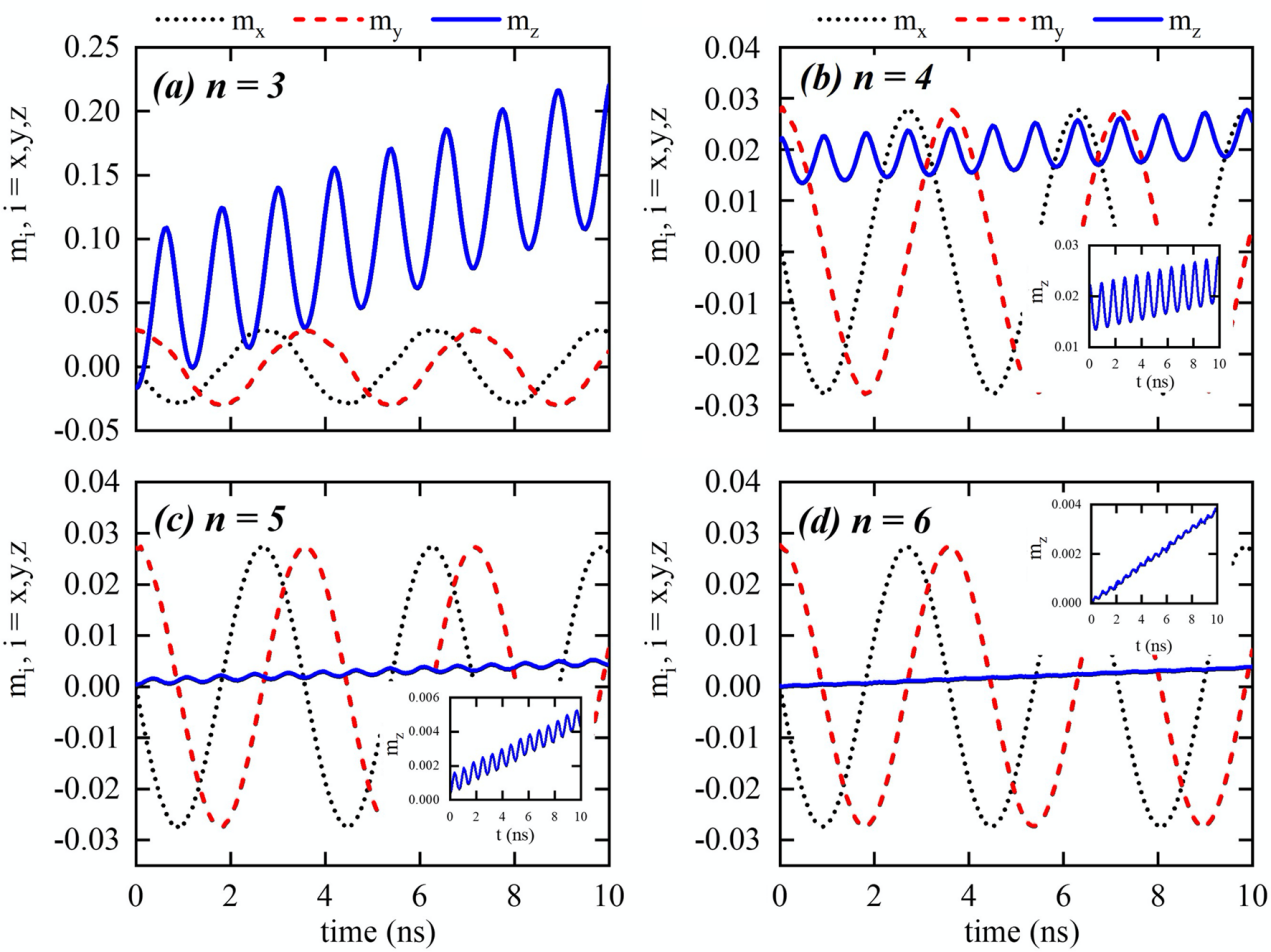
Components of the magnetization as a function of the time for nanowires with different cross-sections with dimensions given by S1. The insets in figures (**b**,**c**,**d**) are the amplification of the component $M_z/M_s$.

**Figure 3:**
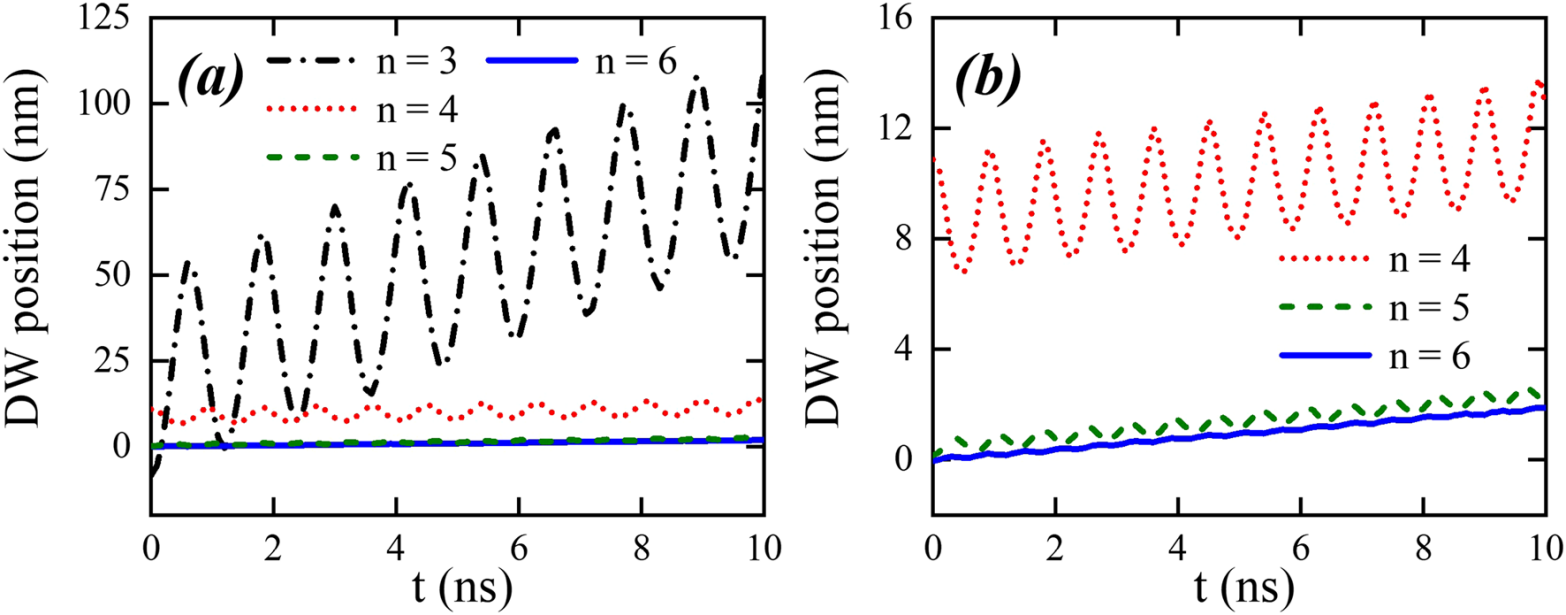
(**a**) DW position as a function of time, for nanowires with different cross-sections with dimensions given by S1. *n* is the number of sides of the nanowire cross-section. (**b**) Zoom for the DW position along nanowires with $n=4$, $n=5$, and $n=6$.

Starting from this state, we apply a constant magnetic field of 10 mT along the $z+$ direction during 10 ns, to determine the influence of the cross-section shape on the DW dynamics. The time dependence of the magnetization for the S1 wires, as obtained by micromagnetic modelling, is displayed in Fig. 2, where the label *n* represents the number of sides of the polygon and, as it will be shown after, it is also related to the number of easy directions of the nanowire cross-section. The component $m_z$ is related to the DW position, and the components $m_x$ and $m_y$ describe the DW phase. During its motion, we observe a DW rotation around the nanowire axis, while displacing along the *z*-axis direction with an oscillatory behavior. An interesting result is that the frequency of the DW rotation around the z-axis is shape-independent, being the same as that in a cylindrical nanowire under equivalent conditions^38^. This result agrees with those obtained for straight and bent nanowires with cylindrical cross-section^34,36,38–41^, which revealed that once the DW is displacing in the Walker regime, the rotation frequency is a function of the external magnetic field and current density. On the other side, the frequency and amplitude of the DW oscillatory motion along the z-axis, are strongly shape dependent. Indeed, Fig. 3 reveals that a DW propagating along a nanowire with a cross-section given by a polygon of *n* sides oscillates *n* times during one rotation. Additionally, one can notice that the oscillation amplitude is inversely proportional to *n*, being very small in the HNW. These aspects of the DW dynamics in multifaceted nanowires can be understood from the fact that the DW rotation is affected just by the external magnetic field, which is pointing along the *z*-axis direction. That is, the magnetic field produces a torque in the *xy*-plane, which makes the DW to rotate, producing changes in the exchange and dipolar interactions. Therefore, the oscillatory motion is governed by changes in the dipolar and exchange effective fields, which are strongly dependent on *n*. Thus, while the external magnetic field plays a role on the rotation motion, changes in the dipolar and exchange effective fields when the DW rotates around the *z*-axis direction affect the DW motion along the wire axis, according to the Landau–Lifshitz equation^45^. In conclusion, the rotational motion does not depend on *n*, but the oscillatory motion in the *z*-axis direction depends on it.

**Figure 4:**
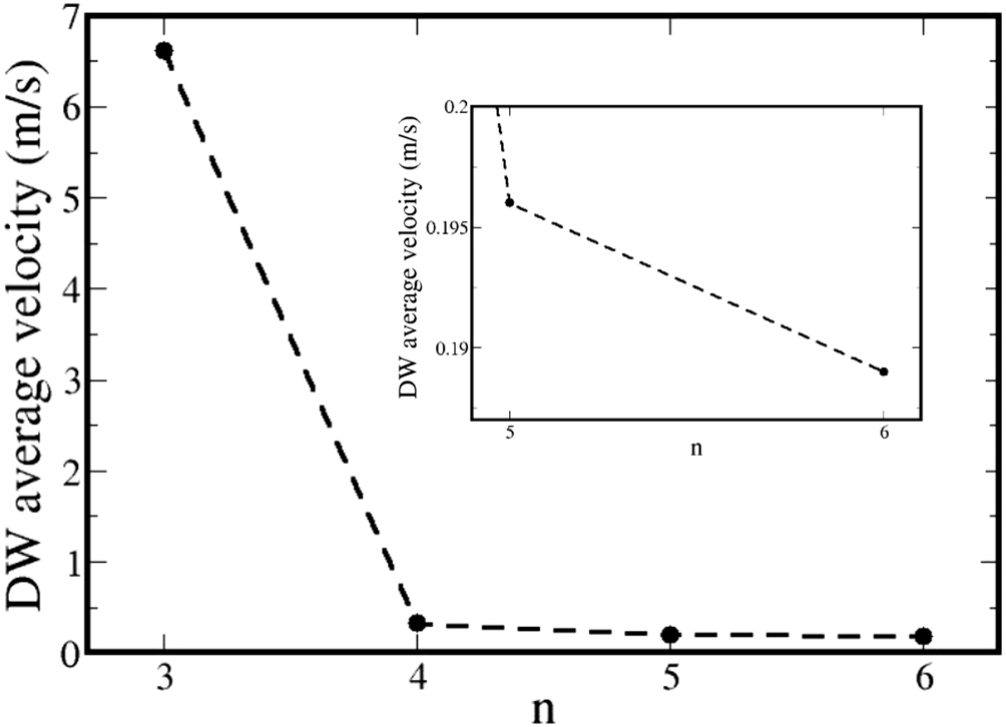
Dots represent the DW average velocity as a function of *n*. The inset illustrates of a zoom of the region between $n=5$ and $n=6$. Black-dashed line is a guide to the eyes.

To be more precise, Fig. 3 illustrates the DW position as a function of time for each of the considered cross-sections. To determine the DW position, we use the following procedure: first, we extract from our results the normalized magnetization, $\mathbf{m} (z)=\mathcal {M}/M_S$, along the nanowire axis for each time step ($\sim$ ps). After that, we fit the obtained magnetization configuration to the well-known transverse DW profile $m(z)=\tanh \left[ (z-z_0)\pi /\Delta _{DW}\right]$, where $z_0$ and $\Delta _{DW}$ are respectively the DW position and width for each time step. Thus, $z_0$ is calculated for each time step. Effectively, previous conclusions become more clear, i.e., both frequency and amplitude of the oscillatory motion strongly depend on the geometry. Furthermore, the domain wall velocity also depends on *n*, and the higher is the *n* value, the smaller is the velocity, recovering analytical predictions for cylindrical nanowires when increasing *n*. Indeed, for the HNW case the oscillation amplitude is small, and its velocity is matching with a cylindrical one. In this way, in cylindrical wires DW propagates slower than in wires of any other geometry. From the DW position, we can determine the DW average velocity $\langle v\rangle =d/t_1$ for each considered nanowire. Here, *d* is the distance that the DW has displaced after $t_1=3.6$ ns (a complete rotation of the DW around *z*-axis). Figure 4 shows the obtained values to $\langle v\rangle$ as a function of *n*. One can notice that this figure depicts the behavior observed in Fig. 3, in which $\langle v\rangle _3\approx 20\, \langle v\rangle _4$, where $\langle v\rangle _n$ is the average velocity for a DW displacing in a nanowire with cross-section of *n* sides. The inset in Fig. 4 presents a zoom in the region between $n=5$ and $n=6$. In this case, we have that $\langle v\rangle _5\approx 1.04\, \langle v\rangle _6$, evidencing that the larger the *n*, the lower the difference between $\langle v\rangle _{n}$ and $\langle v\rangle _{n+1}$.

**Figure 5:**
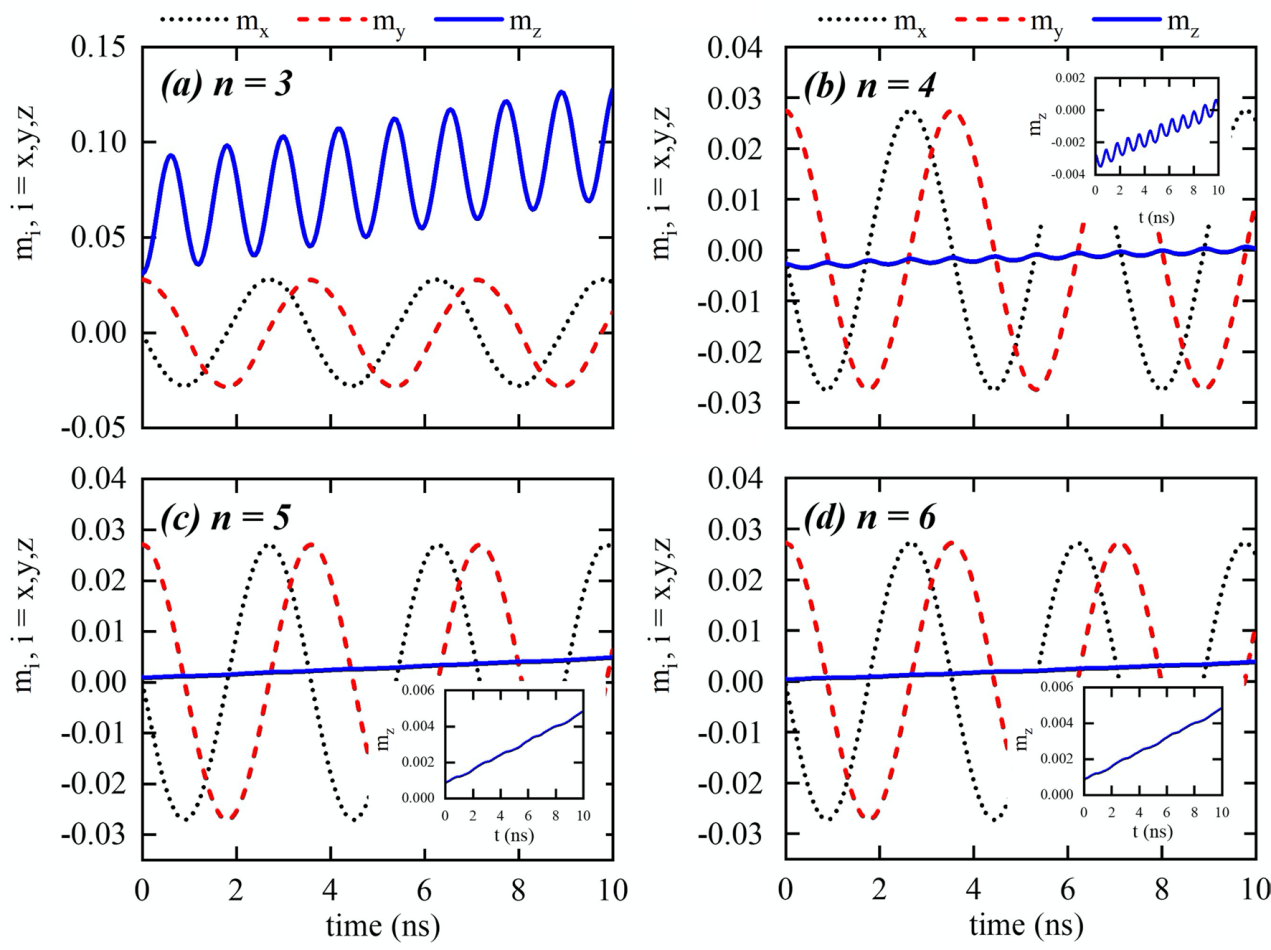
Components of the magnetization as a function of time for nanowires from S2 with different cross-sections. The inset in figures (**b**–**d**) are the amplifications of the component $M_z/M_s$.

To better understand the influence of nanowire faceting on the DW dynamics, we consider a second set of sizes, S2, (see Fig. 1b), with polygons with a smaller cross section area of approximately 360 nm$^2$. The main results regarding the magnetization dynamics are presented in Fig. 5. It can be observed that for the rotation motion, we get the same results as for wires from set S1. However, the difference is observed in the oscillatory behavior along the axis. The amplitude of the oscillations is smaller in all cases. Indeed, in contrast with S1 simulations, oscillations have disappeared both for the PNW and HNW. Furthermore, micromagnetic simulations for smaller structures revealed that this tendency continues, i.e., the smaller is the cross-section, the smaller is the oscillation amplitude, disappearing for all the structures at a specific value of side size. In our calculations, different fields were considered, showing the same qualitative results, with a slower propagations of the DW under lower magnetic fields, as well as lower rotation frequencies.

Aiming at understanding why the oscillations disappear for very thin polygons, independently of the cross-section shape, we performed analytical calculations using the general kinetic momentum theorem, in which it is possible to relate the components of the torque produced on the magnetization, $\varvec{\Gamma }_\theta$ and $\varvec{\Gamma }_\phi$, and their associated rotation velocities $\partial {\theta }/\partial t$ and $\partial {\phi }/\partial t$, that is

1 $$\begin{aligned} \frac{\partial \theta }{\partial t}=-\frac{\gamma }{M_s}\varvec{\Gamma }_\theta \, \text {and}\frac{\partial \phi }{\partial t}=-\frac{\gamma }{M_s}\varvec{\Gamma }_\phi . \end{aligned}$$

The torques can be written in a spherical basis^36^ by using the transfer matrix

2 $$\begin{aligned} \varvec{\Gamma }_{r,\theta ,\phi }=\left( \begin{array}{ccc} \sin \theta \cos \phi &{} \sin \theta \sin \phi &{}\cos \theta \\ \cos \theta \cos \phi &{} \cos \theta \sin \phi &{} -\sin \theta \\ -\sin \phi &{} \cos \phi &{} 0 \end{array}\right) \varvec{\Gamma }_{x,y,z} , \end{aligned}$$

where $\varvec{\Gamma }_{x,y,z}$ are the torques expressed in Cartesian coordinates. Because the considered nanowires are very thin, we adopt a rigid domain wall model, in which the DW does not change its width or shape during is displacement, we do not consider the torque coming from the exchange energy effective field^36^. Therefore, in our calculations we include only the torques produced by the Zeeman and dipolar fields, and the effective damping term. The DW velocity is determined from the fact that the velocity *v* of any spin embedded in a Bloch wall is related to its characteristic width $\Delta$ by $v = -{\Delta \,{\partial _t\theta }}/{\sin \theta }$.

In our analysis the magnetic body is a wire with length $\ell =2L$, with a polygonal cross-section consisting of *n* sides, each of size 2*a*. By considering that the wire cross-section is circunscribed by a cylinder of radius *r*, we have that $a=r\,\cos [\pi (n-2)/2n]$ and consequently, *a* decreases when the number of sides increases in such a way that $a\rightarrow 0$ for $n\rightarrow \infty$ (cylindrical cross-section). Furthermore, from this assumption, we can determine the distance between the surface *S*1 and the geometrical center of the wire, $h=r\,\sin [\pi (n-2)/2n]$ and then, $h\rightarrow r$ for $n\rightarrow \infty$.

The torques produced by Zeeman and damping terms have already been obtained in Ref.^36^. On the other hand, in this work we are not considering a rectangular-shaped cross-section, and therefore the torque produced by the demagnetizing field will be calculated from the definition of the total demagnetizing tensor $\mathbf {N}$ of a prism with *n* sides. Generally, the point-function demagnetization tensor (PFDT) can be given by^50^

3 $$\begin{aligned} \mathbf {N}_p(\mathbf {r})=\frac{1}{4\pi }\nabla \oint _{s'} \frac{1}{|\mathbf {r}-\mathbf {r}'|}d\mathbf {S}', \end{aligned}$$

where $\mathbf {r}$ and $\mathbf {r}'$ are respectively the field and source point locations, and $\mathbf {S}'$ is the surface enclosing the body. The PFDT has two important properties^50^: (i) the trace of the tensor is equal to 1 inside the body and 0 outside; (ii) the tensor satisfies $N_p^{\mu \nu }=N_p^{\nu \mu }$, being symmetric for a closed surface. In this work we are considering regular prisms with two uniaxial rotational periodicities of order *n* along the *z*-axis and of order two around the *y*-axis (see Fig. 1c). The use of the first property of the point-function demagnetization tensor makes it possible to define the matrix representation of the magnetometric demagnetization tensor as^50^

4 $$\begin{aligned} \mathbf {N}_M=\frac{1}{V}\int _{V}\mathbf {N}_{p}(\mathbf {r}) dV=\left( \begin{array}{ccc} N_{1}&{}0&{}0\\ 0&{}N_1&{}0\\ 0&{}0&{}N_2\end{array}\right) , \end{aligned}$$

where $N_2=1-2\,N_1$ inside the magnetic body, and $N_2=-2\,N_1$ outside the magnetic body.

The demagnetization tensor of a prism can also be given by the sum of the $n+2$ surfaces’ demagnetizing tensors associated with each one of the *n* sides of the considered polygon and the two surfaces on the prism borders^51^. That is, if $\mathbf {N}_{Sj}$ is the demagnetizing tensor of the rectangular surface of the side *j* of the prism, we have that $\mathbf {N}_p(\mathbf {r})=\sum _{j=1}^{n}\mathbf {N}_{Sj}+2\mathbf {N}_{b}$, where $\mathbf {N}_b$ is the demagnetizing tensor of the borders of the prism. By considering a nanowire in which $L\gg a$, we can assume $\mathbf {N}_b=0$. Under this framework, the point-function demagnetizing field will be then defined as $\mathbf {H}_d(\mathbf {r})=-4\pi \mathbf {N}_p(\mathbf {r})\cdot \mathbf {M}$.

Without lost of generality, we will assume that the basis of the wire is in the *xz*-plane, in such a way that *S*1 is the surface whose normal vector points along $-\hat{y}$ (see Fig. 1c). In this context, we obtain

5 $$\begin{aligned} \mathbf {N}_{S1}=\left( \begin{array}{ccc} 0\,{}N_{_{S1}}^{xy}\,{}0\\ 0\,{}N_{_{S1}}^{yy}\,{}0\\ 0\,{}N_{_{S1}}^{zy}\,{}0 \end{array}\right) , \end{aligned}$$

where the elements $N_{_{S1}}^{\mu y}$ are given by^52^

6 $$\begin{aligned} N_{_{S1}}^{\mu y}=\frac{1}{4\pi }\int _{-L}^{L}dz'\int _{-a}^{a}dx'\, \frac{\mu -\mu '}{\rho ^{3/2}}\,, \end{aligned}$$

with $\mu =(x,y,z)$, and $\rho =(x-x')^2+(y-y')^2+(z-z')^2$. Because the prism has rotational periodicity about the *y*-axis, it has zero shape anisotropy in the directions transverse to this axis^50^, and we can state that the average of $N^{xy}_{S1}$ and $N^{zy}_{S1}$ over the whole volume of the prism vanishes^50,51^.

The demagnetizing tensor $\mathbf {N}_{Sj}$ of an arbitrary side *j* can be obtained from $\mathbf {N}_{Sj}=\mathbf {R}^{-1}_j,\mathbf {N}_{S1},\mathbf {R}_j$, where $\mathbf {R}_{j}$ is the transformation matrix defining a rotation along *z*-axis,

7 $$\begin{aligned} \mathbf {R}_{j}=\left( \begin{array}{ccc} \cos \varphi _j&{}\sin \varphi _j&{}0\\ -\sin \varphi _j&{}\cos \varphi _j&{}0\\ 0&{}0&{}1 \end{array}\right) , \end{aligned}$$

where $\varphi _j=2\pi (j-1)/n$. These assumptions allow us to determine the point-function demagnetizing tensor, evaluated as

8 $$\begin{aligned} \mathbf {N}_p(\mathbf {r})=\sum _{j=1}^{n}\mathbf {N}_{Sj} =\left( \begin{array}{ccc} \frac{n }{2}\,N_y&{}0&{}0\\ 0&{}\frac{n}{2}\,N_y&{}0\\ 0&{}0&{}1-n\,N_y \end{array}\right) , \end{aligned}$$

where $N_y=V^{-1}\int N_{_{S1}}^{yy}\,dV$. The calculation of this integral for an arbitrary geometry is very hard. Nevertheless, from the properties of the demagnetizing tensor, we can determine $N_y$. Indeed, since the nanowire is considered infinite because $L\gg a$, we have that $1-n\,N_y=0\Rightarrow N_y=1/n$. This result is in agreement with the theorem that states that if a sample has uniaxial rotational periodicity $n \ge 3$, then it has zero-shape anisotropy along the directions transverse to that axis^50^. Due to this zero-shape anisotropy, one can notice that in the approximation of infinitely thin and long nanowire, the demagnetizing tensor components along the x and y directions of any regular polygon are equal. Then, from the dynamical equations obtained by Mougin et al.^36^, we can state that the dynamics of a rigid DW propagating along a nanowire with cross-section given by a thin regular polygon follows the same behavior observed in a cylindrical nanowire^38^. That is, the dynamics of a DW displacing in thin nanowires is independent of the cross-section geometry, and no Walker regime is observed. Therefore, the velocity of the DW center ($\theta =\pi /2$) is

9 $$\begin{aligned} v=\frac{H\,\alpha \,\Delta }{1+\alpha ^2}. \end{aligned}$$

**Figure 6:**
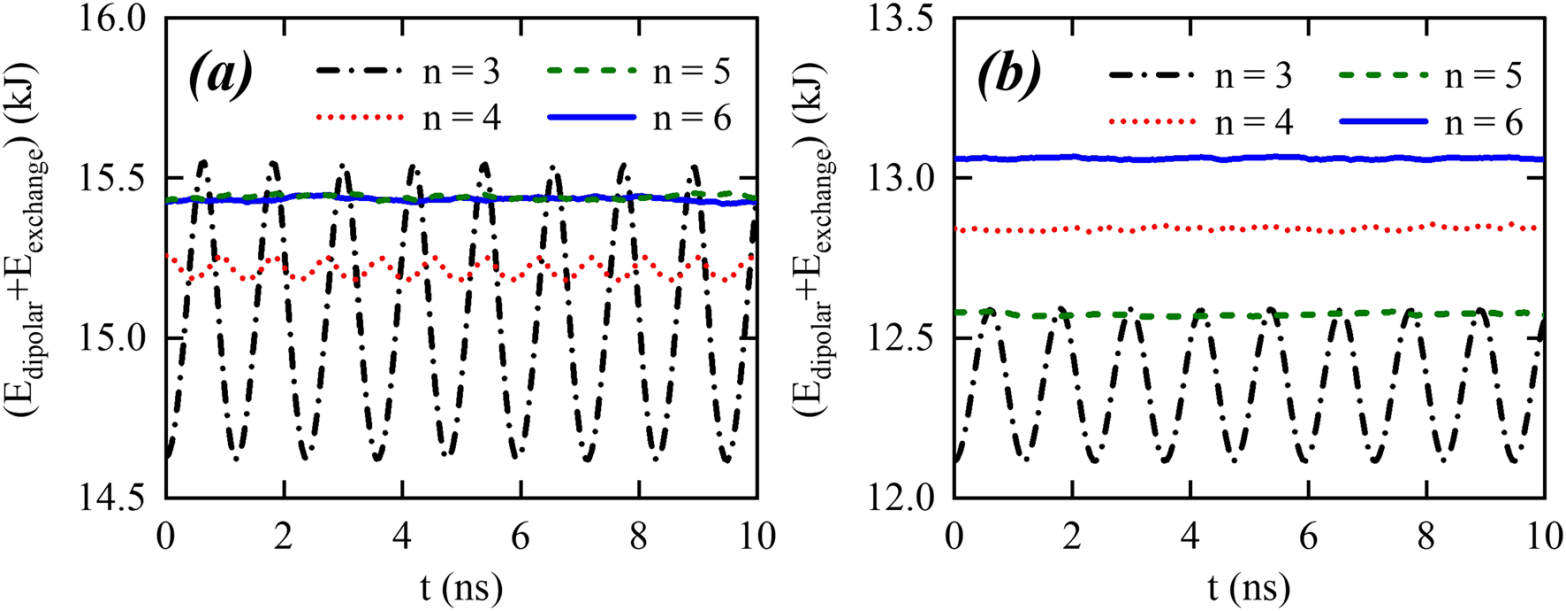
(**a**,**b**) The behavior of the sum of exchange and dipolar energies as a function of time obtained from micromagnetic simulations, for the geometries considered in S1 and S2, respectively.

**Figure 7:**
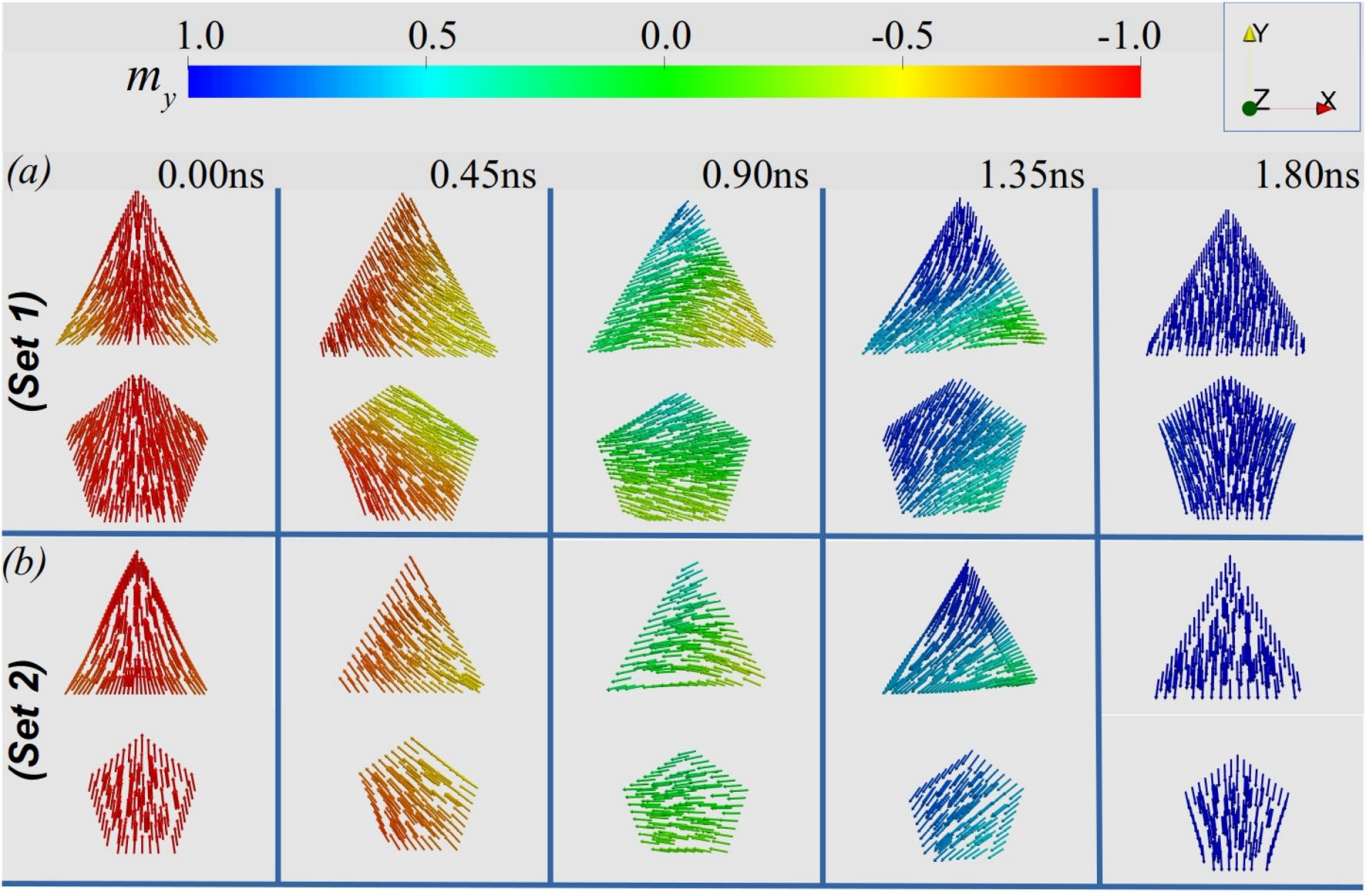
Magnetic configuration of the cross-section corresponding to the $m_y$ component of the DW center for the triangular and pentagonal cases at different times ($t = 0,\, 0.45,\, 0.90,\, 1.35$, and 1.80 ns). (**a**,**b**) Correspond to the geometrical parameters of S1 and S2, respectively.

The above results allow us to conjecture that the oscillations of DW motion, detected by micromagnetic simulations and dependent on the polygon shape, appear as a consequence of the violation of the theoretical assumption of rigid domain wall. Indeed, when the dimensions of the nanowire cross-section diminish, the DW does not present changes in its structure and shape during the rotation around the nanowire cross-section, and then no oscillations appear in its motion. From the set of DW thicknesses shown in Fig. 1b, we conclude that the narrower is the nanowire, the thinner is $\Delta _{\text {DW}}$, approaching the analytical value $\Delta _{\text {DW}}=\pi \sqrt{4A/(\mu _0M_s^2)}\approx 26$ nm, corresponding to an infinite long and narrow nanowire when decreasing the cross section as it does in cylindrical nanowires^53^. The impact of this fact into the DW dynamics is crucial as the DW velocity is proportional to $\Delta _{\text {DW}}$. Effectively, the DW is slower for the S2 ($v=2$ m/s) than for the S1 ($v=6.5$ m/s).

To understand the oscillations observed during the DW motion, we analyze the energy of the DW during its rotation around the nanowire. Our results are depicted in Fig. 6, evidencing that during one rotation the DW energy varies *n* times for wires in set S1. The amplitude of the energy variation diminishes with *n* in such a way that it is practically constant for the HNW. The amplitude of energy changes also decreases with the size of each side, as evidenced in Fig. 6b, where we present the energy of the DW for set S2. Because the magnetic field necessary to ensure that the DW will overtake the energy barrier, starting to rotate around the nanowire cross-section, increases with the energy amplitude, these results allow us to state that the Walker field for which the DW starts its forth and back motion depends on both *n* and the side size of the regular polygon, in such a way that the lower *n*, the higher the Walker limit. Additionally, the greater the side size, the higher the magnetic field generating the oscillatory behavior for the DW motion. The analysis of this figure reveals also that the DW energy in the PNW and HNW is almost constant along a rotation. The changes of the DW energy comes also from changes in the DW width and shape during the rotation around a nanowire, as it is shown in Figs. 1b and 7. Indeed, Fig. 1b evidences the existence of variations in the DW width, which are more pronounced when the DW rotates around the geometries characterized by the S1 system. The amplitudes of the DW width variation ($\Delta w$) are intrinsically related with the DW energy variations and consequently, with the amplitude of the oscillations of the DW position. For instance, one can notice that for the pentagon, $\Delta w_p\approx 1$ nm for S1 and $\Delta w_p\approx 0.2$ nm for S2. Therefore, in the second case, the DW moves almost without changes its width. Additionally, Fig. 7 depicts the snapshots of $m_y$ component of the DW center when it is pointing along different directions during its rotation for the TNW and PNW cases in S1 (*a*) and S2 (*b*) systems. Again, it can be observed that for TNW the shape of the domain wall changes significantly during one rotation, which traduces in changes of the energy. Nevertheless, the DW rotating around a PNW practically does not change its structure (width and shape), and therefore presents a very small variation in its energy. Furthermore, the reduction in the cross-section dimensions lead to smaller changes in the DW structure in such a way that in the PNW of S2, the changes are almost imperceptible in such a way that the DW can be considered as a rigid structure and then there is no Walker regime in this case, as observed in Fig. 5c. We can then conclude that periodic changes in the energy associated with changes in the DW width and shape during its rotation are responsible for the oscillatory motion observed when the DW displaces in nanowires whose side size is large enough to allow structural changes in the DW. Indeed, the DW deformations during its dynamics have previously been suggested as the reason of the Walker breakdown^54^. All the analysis performed from the presented micromagnetic simulations are done for a particular set of magnetic parameters. Nevertheless, since the demagnetizing field is crucial to determine the DW oscillatory motion, we can state that changes in the relation $A/\mu _0M_s^2$ (exchange to dipolar energies) should bring important effects in the DW dynamics. Despite a complete analysis of these effects is out of the scope of this work, we can state that the increase in the $M_s$ value will be associated to a decrease of $\Delta _{\text {DW}}$, with larger amplitudes of the oscillatory motion because the torque exerted by the dipolar effective field would increase. Indeed, it was previously shown that the amplitude of the oscillatory motion of a DW displacing in the Walker regime strongly depends on the energy change during the DW rotation motion^39–41^, i.e., the bigger the changes in the DW energy during a rotation, the bigger the amplitude of the DW motion oscillations. Therefore, because changes in the DW dipolar energy when the DW rotates will be smaller for higher values of $M_s$, the amplitude of the oscillations must be affected. Nevertheless, because the rotation frequency is a function just of the external magnetic field, the oscillation frequency will remain dependent on *n*.

## Conclusions

In conclusion, we analyzed the domain wall dynamics in magnetic nanowires with different cross-sections described by regular polygons. Two regimes for the DW can be observed. The first one is characterized by oscillations along the nanowire, whose frequency depends on the number of sides of the nanowire cross-section. In the second regime no oscillations are observed and DW displaces with a velocity that varies linearly with the magnetic field, independent of the number of sides of the wire polygonal cross-section. In both regimes DW also rotates around the NW. The analysis of the energetics of the DW reveals that changes in the DW shape and structure when it rotates around the nanowire are responsible for changes in the DW energy. The changes in the energy are periodic and depend on the number of sides of the cross-section. It is important to highlight that the propagation velocity of a domain wall in a wire with a triangular cross section is an order of magnitude greater than that of a circular wire. We have also developed an analytical model that describes the rigid DW motion along the considered nanowires, and it was shown that if the DW does not change its structure and shape, its energy is constant and so, no oscillatory motion occurs in this case.
